# The identification and determination of toxin genes of* Vibrio* strains causing hemorrhagic disease on red drum (*Sciaenops ocellatus*) using PCR

**DOI:** 10.1186/s13568-020-01161-w

**Published:** 2021-01-04

**Authors:** Pham Thi Hai Yen, Nguyen Quang Linh, Nguyen Duy Quynh Tram

**Affiliations:** 1 University of Agriculture and Forestry, Hue University, Hue, 530000 Vietnam; 2 Institute of Biotechnology, Hue University, Hue, 530000 Vietnam

**Keywords:** *Sciaenops ocellatus*, TDH, TRH, TLH, ToxR, Toxin gene, *Vibrio*

## Abstract

Data were collected from 30 strains of *Vibrio* and sampled on different organs (brain, hemorrhagic site and digestive tract) of *Sciaenops ocellatus* infection. The results showed that the nucleotide sequences 16S rRNA region are highly similar to those of *V. alginolyticus*, *V. azureus*, *V. fluvialis, V. natriengens* and *V. orientalis,* which were published on Genbank and other, ranging from 98.05 to 100%. The digestive tract has the most common *Vibrio* strains (*V. alginolyticus* [16] *V. azureu*s [7] and *V. fluvialis*). Thereout, 25 of 30 strains of *Vibrio* contained 1 to 3 toxin genes, except *V. parahaemolyticus*. Six parameters were used to measure the DNA polymorphism of 33 homologous DNA sequences in this *Vibrio* bacteria population. The results indicated that number of separate polymorphic sites (S), total number of mutant sites (Eta), number of haplotype (h), haplotype diversity (Hd), average number of nucleotide differences (k), nucleotide diversity (Pi) were 98 (S), 103 (Eta), 9 (h), 0.887 ± 0.032 (Hd), 25.789 (k) and 17.980 × 10–3 ± 0.003 (Pi), respectively (P < 0,05). The G + C content above 1434 sites positions of nucleotide sequences accounted for 0.542. The phylogenetic tree showed that these strains are divided into six groups. As observed, the appearance of isolated *Vibrio* on 3 organs of fish (*S. ocellatus*) hemorrhagic are *V. azureus* (27,67%), *V. alginolyticus* (50%), *V. orientalis* (6,67%) and *V. fluvialis* (16,67%). Through this result, we found that the diversity of *Vibrio* species that appeared on the red drum was used in the 16S rRNA region and the presence of toxin genes in these *Vibrio* species.

## Introduction

More than 100 *Vibrio* spp. have been reported and are predominantly associated with a variety of marine, estuarine, or other aquatic habitats (Janda 2015). Red drum (*Sciaenops ocellatus*) was originally discovered in the Atlantic Ocean and the Gulf of Mexico; it was introduced into China in 1991, and since then, it has been cultured extensively in several provinces in China (Zhang and Sun 2011). In recent years, red drum (*S. ocellatus*) mortalities have been associated with *Streptococcus iniae* infection (Eldar et al. 1999), (Mmanda et al. 2014). Seven *Vibrio* strains (including *V. vulnificus* HM-TA-D2-L2-V2; *V. vulnificus* HM-TA-G2-V1-D2; *V. brasiliensis* HM-X-13/6; *V. cholerae* V-13/6; *V. parahaemolyticus* HM-17/6; *V. cholerae* HM-V-13/6 and *V. vulnificus* HM-X-13/6) causing hemorrhagic disease in red drum (*S. ocellatus*) had only the tlh gene, and none of the *Vibrio* strains had tdh and trh genes (Quang et al. 2020). The research identified this fish (*S. ocellatus*) viperin gene (SoVip) and analyzed its expression in relation to bacterial challenge. The complete SoVip gene is 2570 bp in length and contains six exons and five introns. The open reading frame of 1065 bp is flanked by a 50 untranslated region (UTR) of 34 bp and a 30 UTR of 350 bp and the fish pathogen *Edwardsiella tarda* but is downregulated by the fish pathogens *Listonella anguillarum* and *Streptococcus iniae* (Dang et al. 2010). Toxin gene neutrality was tested by three methods (Tajima’s D test- Methods for sequence analysis of candidate genes obtained; Fu and Li’s D* and F* test, Fu's Fs—statistic P values being significant or not), (Tajima 1989), (Fu and Li 1993) and (Fu 1995), they indicated an excess of low frequency polymorphisms relating to expectation, evidence for a deficiency of alleles, as expected from a recent population bottleneck and the evolution of the studied 30 strains bacteria *Vibrio*, was balancing selection, sudden contraction, rare alleles appeared in populations with low frequency. The studied population had a few individuals showing large differences in comparison with other individuals. The study aims to identify and determine toxin genes in *Vibrio* infected red drum; hence, an understanding of Vibrio spp. infected on fish to cause *Vibriosis* in aquatic animals in brackish and marine water.

## Materials and methods

### Collection of fish disease

**Fig. 1:**
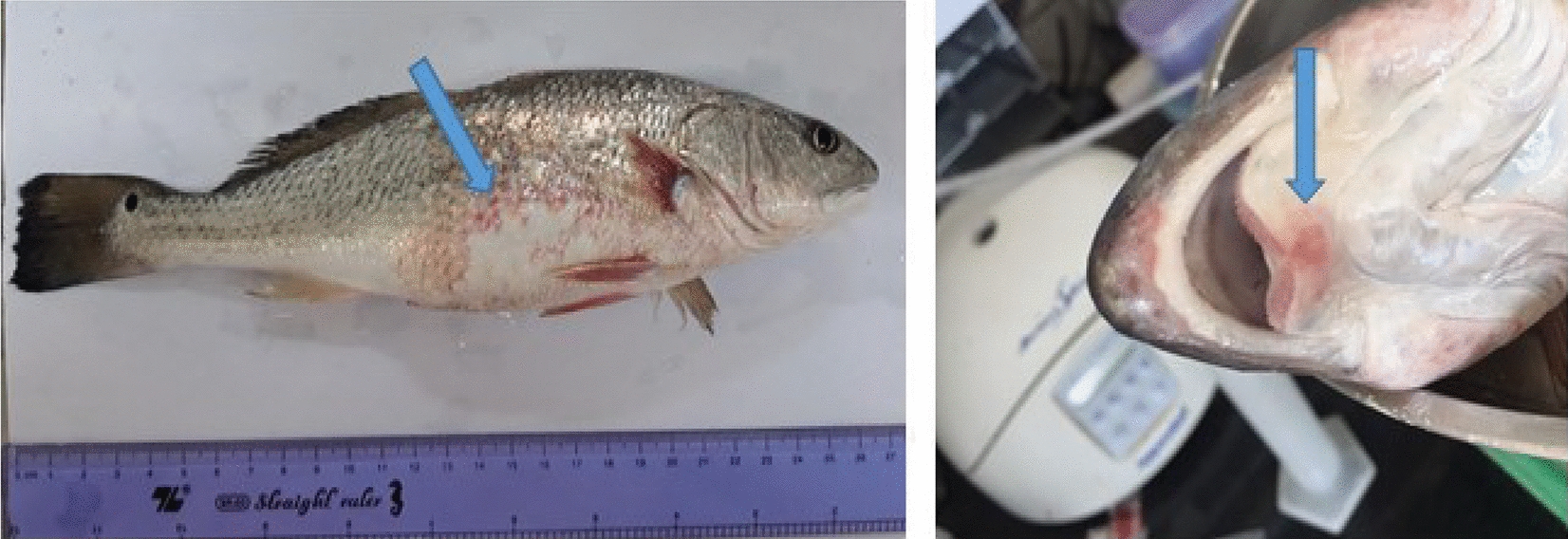
Sample of *Sciaenops ocellatus* hemorrhagic signal

In this study, data were collected from the field and stored at −20 °C, and we used thirty strains of bacteria with different morphologies isolated from three different organs in fish (*S. ocellatus*) that have hemorrhagic disease (Fig. 1) in Thua Thien Hue Province, Vietnam, based on medium TCBS (thiosulphate citrate bile salt sucrose).

### Total DNA extraction method

The DNA extraction method presented in this paper is an improved method of phenol/chloroform extraction according to the method of (Neumann et al. 1992). We eliminated the step to use SDS/lysozyme or proteinase K and extraction of cells directly by phenol. To extract the DNA from bacteria isolated from hemorrhagic disease in fish, a 1 mL cell suspension was centrifuged at 8.000 rpm for 2 min for the collection of pellet cells. After removing the supernatant, the cells were washed with 400 µl STE buffer (100 mM NaCl, 10 mM Tris/HCl, 1 mM EDTA, pH 8.0) twice and then centrifuged at 8000 rpm for 2 min. The pellets were resuspended in 200 µl TE buffer (10 mM Tris/HCl, 1 mM EDTA, pH 8.0). After this, 100 µl Tris-saturated phenol (pH 8.0) was added to these tubes, followed by a vortex-mixing step of 60 s. The samples were subsequently centrifuged at 13.000 rpm for 5 min at 4 °C to separate the aqueous phase from the organic phase. A total of 160 µl of the upper aqueous phase was transferred to a clean 1.5 ml tube. Forty microliters of TE buffer was added to 200 µl, mixed with 100 µl of chloroform and centrifuged for 5 min at 13,000 rpm at 4 °C. The lysate was purified by chloroform extraction until a white interface was no longer present; this procedure might have to be repeated two to three times. A total of 160 µl of the upper aqueous phase was transferred to a clean 1.5 ml tube. Then, 40 µl TE and 5 µl RNase (at 10 mg/ml) were added and incubated at 37 °C for 10 min to digest RNA. Then, 100 µl chloroform was added to the tube, mixed well and centrifuged for 5 min at 13.000 rpm at 4 °C. Then, 150 µl of the upper aqueous phase was transferred to a clean 1.5 ml tube. The aqueous phase contained purified DNA and was directly used for the subsequent experiments or stored at 20 °C. The purity and yield of the DNA were assessed spectrophotometrically by calculating the A_260_/A_280_ ratios and the A_260_ values to determine protein impurities and DNA concentrations according to (Neumann et al. 1992).

### Determination of toxin gene

The presence of toxin genes in *Vibrio* spp. strains was determined through the presence of genes encoding toxic proteins (*tlh, tdh, trh and toxR*), which are based on specific primers for these genes (Table 1). PCR procedure: 50 ng of total DNA, 10 pmol of each primer, 25 µl PCR master mix 2 × (2.4 mM dNTPs each, 0.3 units Taq DNA polymerase, Promega, USA), and sterile distilled water (total volume of 50 µL). PCR amplification was performed in an MJ Mini™ Thermal Cycler (Bio-Rad, USA) as follows: 94 °C for 3 min; followed by 30 cycles at 94 °C for 1 min, 50 °C for 1 min, and 72 °C for 1 min; and a final cycle of 72 °C for 7 min. PCR products were used for electrophoresis on a 1% agarose gel using standard electrophoresis procedures in TAE 1X buffer with ethidium bromide dye, and electrophoresis images were read by a direct UV reading system (UV-transilluminator, Model: DyNa Light).

**Table 1:** Sequence of primers

Genes	Primer names	Nucleotide sequences 5′ → 3’	Size (bp)	References
*toxR*	*toxR*-F	GTCTTCTGACGCAATCGTTG	367	Luan et al. (2007); Marlina et al. (2007)
	*toxR*-R	ATACGAGTGGTTGCTGTCATG		
*Tdh*	*tdh*-F	GTAAAGGTCTCTGACTTTTGGAC	500	Luan et al. (2007); Marlina et al. (2007)
	*tdh*-R	TGGAATAGAACCTTCATCTTCACC		
*Trh*	*trh*-F	TTGGCTTCGATATTTTCAGTATCT	269	Luan et al. (2007); Marlina et al. (2007)
	*trh*-R	CATAACAAACATATGCCCATTTCC		
*Tlh*	*tlh*-F	AAAGCGGATTATGCAGAAGCACTG	450	Luan et al. (2007); Marlina et al. (2007)
	*tlh*-R	GCTACTTTCTAGCATTTTCTCTGC		

*Tdh*, Thermostable direct hemolysin; *trh*, TDH-related hemolysin; *tlh*, Thermolabile hemolysin; *toxR*, Toxin operon (Luan et al. 2007; Marlina et al. 2007)

### 16S rRNA gene amplification and sequencing

PCR was performed to amplify the 16S rRNA region originating from the genome with a pair of 16S primers: 27F: AGAGTTTGATCMTGGCTCAG and 1492R: TACGGYTACCTTGTTACGACTT (Jeremy A Frank et al. 2008). PCR was performed on Applied Biosystems—Life Technologies—Thermo Fisher Scientific—USA with a reaction component of 25 µl PCR master mix 2 × (2.4 mM dNTPs each, 0.3 units Taq DNA polymerase), 10 pmol of 27F primer, 10 pmol of 1492 primer, 1 µl of total DNA (50 ng/µl) and sterile distilled water to a final volume of 50 µl. The 16S rRNA gene region was amplified with the following thermal cycle: 95 °C/5 min; 30 cycles × (95 °C/60 s; 57 °C/50 s; 72 °C/60 s); 72 °C/10 min. Aliquots (10 µl) of PCR products were electrophoresed and visualized in 1% agarose gels using standard electrophoresis procedures in TAE 1X buffer with ethidium bromide dye, and electrophoresis images were read by a direct UV reading system (UV transilluminator, Model: DyNa Light). Partial 16S rRNA genes of selected isolates in each site were sequenced by MACROGEN, Republic of Korea (dna.macrogen.com). Finally, the 16S rRNA sequence of the isolation was compared with that of other microorganisms using BLAST (http://www.ncbi.nlm.nih.gov/BLAST/Blast.cgi).

### Sequencing and analyzing genetic relationships

The PCR products of the 16S rRNA region were purified with Isolate II PCR and Gel (Bioline) kits. Then, they were sequenced directly by the dideoxy termination method on the ABI PRISM® 3100 Avant Genetic Analyzer (Applied Biosystems) at Maccrogen Company, Korea (dna.macrogen.com). The nucleotide sequences were arranged based on the Clustals program (Thompson et al. 1997) and edited by using BioEdit 7.0.5 software (Hall 1999). Finally, the 16S rRNA sequence of the isolation was compared with that of other microorganisms using BLAST (http://www.ncbi.nlm.nih.gov/BLAST/Blast.cgi). The DNA polymorphism analysis was based on eight parameters, including the number of separate polymorphic sites (S), total number of mutant sites (Eta), number of haplotypes (h), haplotype diversity (Hd), average number of nucleotide differences (k), and nucleotide diversity (Pi), considered polymorphic measurements in the population (Rozas et al. 2005). Neutrality was tested based on three methods, Tajima’s D test (Tajima 1989), Fu and Li’s D * and F* test (Fu and Li 1993) and Fu's (Fu 1995), using DNASP 6.0 software. A phylogenetic tree showing genetic relationships was built by MEGA X software (Molecular Evolution Genetics Analysis) based on the UPGMA method (Sneath and Sokal 1973). The optimal tree with the sum of branch length equal to 0.08795656 is shown. The percentage of replicate trees in which the associated taxa clustered together in the bootstrap test (1000 replicates) is shown next to the branches (J 1985). The tree was drawn to scale, with branch lengths in the same units as those of the evolutionary distances used to infer the phylogenetic tree. The evolutionary distances were computed using the maximum composite likelihood method (Tamura et al. 2004) and are in units of the number of base substitutions per site. This analysis involved 48 nucleotide sequences. All ambiguous positions were removed for each sequence pair (pairwise deletion option). There were a total of 1434 positions in the final dataset. Evolutionary analyses were conducted in MEGA X (Kumar et al. 2018).

## Results

### PCR result

The PCR products of the 16S rRNA region were purified with Isolate II PCR and Gel (Bioline) kits. Then, they were sequenced directly by the dideoxy terminator method on the ABI PRISM® 3100 Avant Genetic Analyzer (Applied Biosystems) at Maccrogen Company, Korea (dna.macrogen.com). The 16S rRNA region was approximately 1450 bp for the remaining 30 isolated bacterial strains based on TCBS medium. The BLAST result on NCBI was used to verify and compare with the sequences of the *Vibrio* spp. with accession number GenBank (Table 2) showed that the nucleotide sequences obtained were highly similar to those of *V. alginolyticus, V. azureus, V. fluvialis* and *V. orientalis*, ranging from 98,05 to 100% (Table 2).

**Table 2:** Phylogenetic affiliation of isolates on the basis of 16S rRNA gene sequences by using the BLAST program in the GenBank database based on sequence similarity and determination of toxin genes

No	Isolated	Genbank code	GenBank reference	Similarity (%)	Genes			
					*toxR*	*tdh*	*trh*	*Tlh*
1	*Vibrio alginolyticus strain* YHTH7	MT953948	MN874162.1	98.06	–	–	+	–
2	*Vibrio azureus strain* YVL11	MT953949	KT986135.1	100	–	–	–	–
3	*Vibrio azureus strain* HTH12	MT953950	KT986135.1	100	–	–	–	–
4	*Vibrio alginolyticus strain* YN14	MT953951	MH298564.1	98.05	+	–	–	+
5	*Vibrio fluvialis strain* YHTH16	MT953952	CP051126.1	100	+	–	–	+
6	*Vibrio alginolyticus strain* YVL22	MT953953	MN843961.1	99.72	+	–	+	+
7	*Vibrio alginolyticus strain* YVL24	MT953954	MN938185.1	99.86	+	+	–	+
8	*Vibrio alginolyticus strain* YVL26	MT953955	CP051109.1	99.59	–	–	–	–
9	*Vibrio orientalis strain* YVL27	MT953956	MN945276.1	100	–	–	–	+
10	*Vibrio alginolyticus strain* YN34	MT953957	MN938360.1	99.65	–	–	+	+
11	*Vibrio azureus strain* YVL5	MT953958	KT986135.1	100	–	–	+	–
12	*Vibrio azureus strain* HTH6	MT953959	KT986135.1	100	+	–	–	+
13	*Vibrio fluvialis strain* YHTH18	MT953960	CP051126.1	100	+	–	+	–
14	*Vibrio alginolyticus strain* YN19	MT953961	MH298564.1	98.05	+	–	–	+
15	*Vibrio alginolyticus strain* YN29	MT953962	MN843961.1	99.72	–	–	–	+
16	*Vibrio alginolyticus strain* YVL31	MT953963	MN938185.1	99.86	–	–	+	+
17	*Vibrio alginolyticus strain* YVL40	MT953964	CP051109.1	99.59	+	–	–	+
18	*Vibrio alginolyticus strain* YVL43	MT953965	MN938360.1	99.65	+	–	–	–
19	*Vibrio orientalis strain* YVL42	MT953966	MN945276.1	100	+	–	+	+
20	*Vibrio alginolyticus strain* YHTH44	MT953967	MN874162.1	98.06	+	–	+	+
21	*Vibrio azureus strain* YVL45	MT953968	KT986135.1	100	+	–	–	+
22	*Vibrio azureus strain* YVL46	MT953969	KT986135.1	100	–	–	+	–
23	*Vibrio fluvialis strain* YHTH47	MT953970	CP051126.1	100	–	–	–	–
24	*Vibrio azureus strain* YVL33	MT953971	KT986135.1	100	+	+	–	–
25	*Vibrio azureus strain* YHTH35	MT953972	KT986135.1	100	–	–	–	+
26	*Vibrio fluvialis strain* YHTH37	MT953973	CP051126.1	100	–	–	–	+
27	*Vibrio alginolyticus strain* YN38	MT953974	MH298564.1	98.05	+	–	–	+
28	*Vibrio alginolyticus strain* YVL84	MT953975	MN843961.1	99.72	–	–	–	–
29	*Vibrio alginolyticus strain* YVL85	MT953976	MN938185.1	99.86	–	–	–	+
30	*Vibrio alginolyticus strain* YVL86	MT953977	CP051109.1	99.59	+	–	–	–

*Tdh*, Thermostable direct hemolysin; *trh*, TDH-related hemolysin; *tlh*, Thermolabile hemolysin; *toxR*, Toxin operon

GenBank registration Code No: MT953948[ACCN]: MT953977[ACCN]; https://submit.ncbi.nlm.nih.gov/subs/?search=SUB8088865

**Fig. 2:**
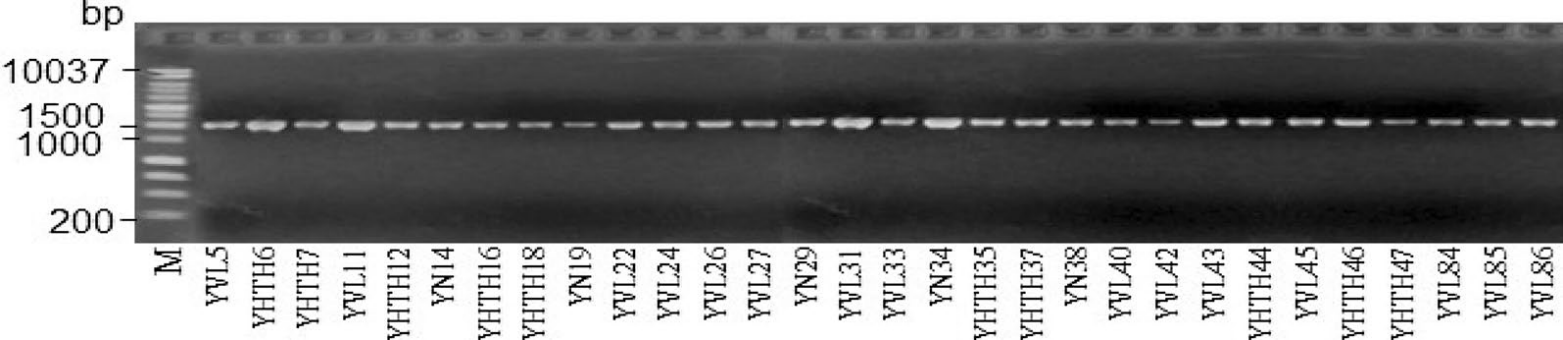
Electrophoresis of PCR product. M: DNA mass scale (HyperLadder™ 1 kb (200 bp to 10,037 bp), Bioline, Meridian Bioscience

The results indicated that all PCR products of the 16S rRNA region in the 30 isolated bacterial strains based on medium TCBS showed a single band with a 100% amplification rate. All samples gave high DNA concentrations and are clearly seen. The obtained size was approximately 1.500 bp, which is in line with the initial expected size (Fig. 2).

### Determination of toxin gene

The agarose gel electrophoresis of PCR products determined the presence of the *trh*, *tdh*, *tlh* and *toxR* genes at bands 269 bp, 500 bp, 450 bp and 367 bp, respectively (Fig. 2). We found that 25/30 strains of *Vibrio* contained at least 1 toxic gene, whereas 5 isolates carried 3 toxin genes. However, none of these isolates consisted of all virulence toxin genes (Table 2). The results clearly indicated the presence of virulence toxins (*trh, tdh and tlh*) and a regulator toxin (*toxR*). Among them, 18 isolates presented *tlh,* while only 2 isolates were found to carry *tdh* gen.

### Sequencing and analyzing genetic relationships

Six parameters, including the number of polymorphic sites (S), total number of mutant sites (Eta), number of haplotypes (h), haplotype diversity (Hd), average number of nucleotide differences (k), and nucleotide diversity (Pi), were used to evaluate the diversity of the 30 studied *Vibrio* strains. As shown in Table 4, ninety-eight separate polymorphic positions (S) created 103 mutant positions (Eta) shown in 30 studied bacterial strains. *Vibrio* were classified into nine types of haplotypes (h) with a haplotype diversity coefficient accounting for 0.887 ± 0.032 (Hd), an average number of nucleotide differences of 25.789 (k), and a nucleotide diversity coefficient of 17.980 × 10^–3^ ± 0.003 (Pi). All indicators were processed with statistical significance *p* < *0,05.* The G + C content above 1434 site positions of nucleotide sequences accounted for 0.542 (Table 3). Three methods, namely, Tajima’s D test, Fu and Li’s D* and F* test, and Fu's Fs, were used to test neutrality.

**Table 3:** DNA diversity based on the 16S rRNA region of the bacterial *Vibrio* population using the DNASp 5.0 program (Rozas and Rozas 2005)

Genetic region	S	Eta	H	G + C content (1434 sites)	Hd	K	Pi (× 10^−3^)
*16S rRNA*	98	103	9	0.542	0.887 ± 0.032	25.789	17.980 ± 0.003

S, Number of variable sites; Eta, Total number of mutations; H, Number of haplotypes; Hd, haplotype (gene) diversity; Pi, nucleotide diversity (per site); k, average number of nucleotide differences

**Fig. 3:**
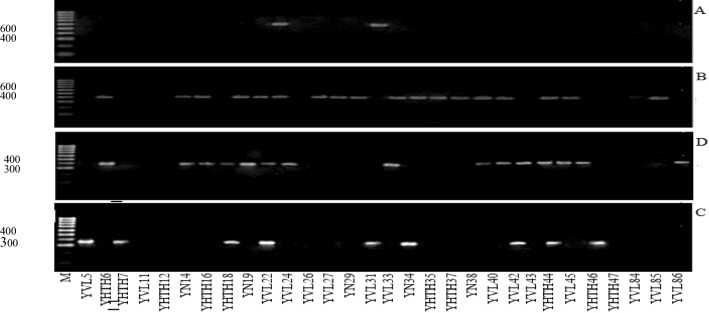
Electrophoresis determination of toxin gene. M: DNA mass scale (100 bp to 1000 bp), Biobase, Meridian Bioscience). **a** PCR product of gene *tdh*. **b** PCR product of gene *tlh*. **c** PCR product of the *toxR gene* and **d** PCR product of the *trh gene*

The results in Table 4 indicated that a negative Tajima’s D signifies an excess of low-frequency polymorphisms compared with the initial expectation (*statistical significance: not significant*, *p* > *0.10*). Meanwhile, a positive value of F_S_ (13.659) is evidence for a deficiency of alleles, as would be expected from a recent population bottleneck (*Strobeck's S statistic: 0.000*). In addition, Fu and Li's F * (*statistical significance 0.10* > *p* > *0.05*) and the value of Fu and Li's D* (statistical significance: **, P < *0.02*) both yielded positive results, which showed that the evolution of the 30 studied bacterial strains in the *Vibrio* population was balancing selection and sudden contraction; in other words, rare alleles appeared in populations with low frequency, and the studied population had very few individuals showing large differences in comparison with other individuals in the population (Table 4). The phylogenetic tree shows the genetic relationship of thirty *Vibrio* strains isolated from three different parts of the fish (*S. ocellatus*) using the UPGMA method. Figure 3, these strains are divided into six groups. Among these, group I includes the strains of isolated *Vibrio* that are closely related to *V. azureus*. These strains are mainly concentrated in the digestive system and are hemorrhagic. Groups II, III and V consist of *Vibrio* strains isolated in 3 different parts (brain, hemorrhagic and digestive system). They are closely related to *V. alginolyticus*. Group 4 includes two strains isolated from the ulcer that are closely related to *Vibrio orientalis*. Group VI consists of 4 strains, concentrated in the digestive system and having a close genetic relationship with *V. fluvialis* (Fig. 3). As observed, the isolated *Vibrio* on 3 organs of red drum fish showing signs of hemorrhagic bleeding were *V. azureus* (27,67%), *V. alginolyticus* (50%), *V. orientalis* (6,67%) and *V. fluvialis* (16,67%).

**Table 4:** Neutrality test results based on the 16S rRNA region of the bacterial *Vibrio* population

Genetic region	Tajima’s D test	Fu and Li’s D* test	Fu and Li’s F* test*	Fu's Fs
*16S rRNA*	−0.03099	1.91401	1.49643	13.659
	Statistical significance: not significant, P > 0.10	Statistical significance: **, P < 0.02	Statistical significance: Not significant, 0.10 > P > 0.05	Strobeck's S statistic: 0.000

## Discussion

In this study, we isolated 30 strains of *Vibrio* from three different organs (brain, hemorrhagic site and digestive tract) of *S. ocellatus*. The results showed that the nucleotide sequence 16S rRNA regions are highly similar to those of *V. alginolyticus*, *V. azureus*, *V. fluvialis* and *V. orientalis* published in GenBank, ranging from 98.05 to 100%. The digestive system has the most common *Vibrio* species (*V. alginolyticus, V. azureus*). None of the *V. parahaemolyticus* species were present in the samples, and the same report identified that *Vibrio* species were isolated from cultured olive flounder (*Paralichthys olivaceus*) on Jeju Island, South Korea, and none of the *V. parahaemolyticus* species were also present (Sohn et al. 2019), as reported by (Quang et al. 2020). The *V. parahaemolyticus* strain is a pathogen causing shrimp disease, (Dao et al. 2014), (Khanh et al. 2019).

The presence of toxic genes related to the hemolysin of fish was found in various *Vibrio* sp. Meanwhile, approximately 50% of isolates consisted of toxin operon genes. All *V. parahaemolyticus* isolates contained the *toxR* genes, but the *trh* gene did not exist in clams (*Corbicula moltkiana*) (Marlina et al. 2007). Our data confirmed that three isolates carried both *toxR* and *trh* genes, including isolates that exhibited high similarity to *V. fluvialis*, *V. alginolyticus*, and *V. orientalis. TDH* is an enzyme that lyses human red blood cells on Wagatsuma blood agar plates, which is referred to as the Kanagawa phenomenon positive. Another toxin produced by Kanagawa phenomenon-negative *Vibrio* strains is the tdh-related hemolysin (*trh*) toxin encoded by the trh gene (Al-Othrubi et al. 2011). Thermolabile hemolysin (*tlh*) is another *Vibrio* enterotoxin that causes blood cell lysis in infected fish, and *tlh* is encoded by the tlh gene (Hasrimi et al. 2018). Among the 4 toxin genes (*toxR*, *tdh, trh* and *tlh*) investigated from *Vibrio* spp. causing hemorrhagic disease in *S. ocellatus*, the results showed that the frequency of the *toxR* gene was detected in the 15 isolates using PCR assay, lowest of *tdh* gene was 2 isolates, *trh* gene was 9 isolates and the highest of *tlh* was 18 isolates using PCR assay. In addition, the frequency of toxin gene occurrence also showed that there were 5/30 *Vibrio* strains none carrying the toxin gene (code number: YHTH12; YHTH47; YVL11; YVL26 and YVL84), 10/30 strains carrying only 1 toxin gene, 11/30 strains carrying 2 toxin genes and 4 strains carrying 3 toxin genes (*V. alginolyticus strain 3–31*, code number YHTH44 (toxR, trh and tlh); *V. alginolyticus strain 3–31*, code number YVL22 (*toxR, trh* and *tlh*); *V. alginolyticus strain 3–5*, code number YVL24 (*toxR*, *tdh* and *tlh*) and *V. orientalis strain 5–13*, code number YVL42 (*toxR*, *trh* and *tlh)*). None of *Vibrio* carried all 4 toxin genes. All *Vibrio* strains isolated from three marine fish species (*S. ocellatus*, *Lates calcarifer* and *Epinephelus fuscoguttatus*) carried only one *tlh* gene (Quang et al. 2020). According to Long et al. 2019, we isolated and identified the *V. parahaemolyticus* 01 strain in Thua Thien Hue Province, Vietnam, causing ulcer disease in *S. ocellatus*. The full-length thermolabile hemolysin (tlh) gene (1257 bp), encoding the antigen thermolabile hemolysin toxin (*tlh*) of *Vibrio* sp. was cloned and sequenced successfully. Sequence analysis of cloned genes showed complete similarity to the *V. parahaemolyticus* strain (GenBank: AY289609.1) (Long et al. 2019). We further examined the presence of virulence genes homologous to those in *V. cholerae* (*toxR, toxS, VPI* and ace); *toxR* was found in 16 V*. alginolyticus* strains, and *toxS* was found in 17 strains out of 34. Indicated in two species (*Dicentrarchus labrax*) and (*Sparus aurata*). Positive amplification of the virulence pathogenicity island (VPI) was produced by 12 V*. alginolyticus* strains. Finally, the expected amplification fragment was found in 7 V*. alginolyticus* isolates. Thus, the pathogenicity of *V. alginolyticus* may be the result of a combination of all these factors (Kahla-Nakbi et al. 2009).

Six parameters were used to evaluate the diversity of the 30 studied *Vibrio* bacterial strains. The results showed that ninety-eight separate polymorphic positions (S) created 103 mutant positions (Eta) shown in 30 studied *Vibrio* strains classified into nine types of haplotypes (h) with a haplotype diversity coefficient accounting for 0.887 ± 0.032 (Hd), an average number of nucleotide differences of 25.789 (k), and a nucleotide diversity coefficient of 17.980 × 10^–3^ ± 0.003 (Pi). All indicators were processed with statistical significance *p* < *0.05*. The G + C content above 1434 site positions of nucleotide sequences accounted for 0.542. Neutrality was tested based on three methods (Tajima’s D test, Fu and Li’s D* and F* test, Fu's Fs) showing that there was an excess of low frequency polymorphisms relative to expectation, evidence for a deficiency of alleles, as would be expected from a recent population bottleneck and the evolution of the studied 30 *Vibrio* bacteria population was balancing selection, sudden contraction or in other words, rare alleles appeared in populations with low frequency, the studied population had very few individuals showing large differences in comparison with other individuals in the population. The phylogenetic tree showed the genetic relationship of 30 *Vibrio* strains using the UPGMA method (bootstrap = 1000) and showed that these strains were divided into six groups. As observed, the isolated *Vibrio* on 3 hemorrhagic organs of fish (*S. ocellatus*) were *V. azureus* (27,67%), *V. alginolyticus* (50%), *V. orientalis* (6,67%) and *V. fluvialis* (16,67%).

## Data Availability

All the data were presented in the main paper.

## Abbreviations

PCR: Polymerase chain reaction
TCBS: Thiosulphate citrate bile salt sucrose
tlh: Thermolabile hemolysin toxin
trh: Tdh-related hemolysin
tdh: Thermostable direct hemolysin
toxR: Toxin operon
